# Uterotonic use immediately following birth: using a novel methodology to estimate population coverage in four countries

**DOI:** 10.1186/s12913-014-0667-1

**Published:** 2015-01-22

**Authors:** Jim Ricca, Vikas Dwivedi, John Varallo, Gajendra Singh, Suranjeen Prasad Pallipamula, Nazir Amade, Maria de Luz Vaz, Dustan Bishanga, Marya Plotkin, Bushra Al-Makaleh, Stephanie Suhowatsky, Jeffrey Michael Smith

**Affiliations:** Maternal and Child Survival Program, Jhpiego, 1776 Massachusetts Ave., NW #300, Washington, DC 20036 USA; Maternal and Child Survival Program, JSI Research & Training Institute, Inc, 1776 Massachusetts Ave., NW #300, Washington, DC 20036 USA; Jhpiego, 221, Okhla Phase III, New Delhi, 110 020 India; Maternal Child Integrated Program (MCHIP), Jhpiego, Government Vaccine Institute Campus, Namkum, Ranchi, Jharkhand 834010 India; Director of Maternal and Child Health, Ministry of Health, Maputo, Mozambique; MCHIP, Jhpiego, Maputo, Mozambique; MCHIP, Jhpiego, PO Box 9170, Dar es Salaam, Tanzania; Jhpiego Tanzania, PO Box 9170, Dar es Salaam, Tanzania; MCHIP, Yemen, Haddah Street, Sana’a City, Yemen

**Keywords:** Uterotonic, Estimation, Postpartum hemorrhage (PPH), Delphi method, UUIFB

## Abstract

**Background:**

Postpartum hemorrhage (PPH) is the leading cause of maternal mortality in developing countries. While incidence of PPH can be dramatically reduced by uterotonic use immediately following birth (UUIFB) in both community and facility settings, national coverage estimates are rare. Most national health systems have no indicator to track this, and community-based measurements are even more scarce. To fill this information gap, a methodology for estimating national coverage for UUIFB was developed and piloted in four settings.

**Methods:**

The rapid estimation methodology consisted of convening a group of national technical experts and using the Delphi method to come to consensus on key data elements that were applied to a simple algorithm, generating a non-precise national estimate of coverage of UUIFB. Data elements needed for the calculation were the distribution of births by location and estimates of UUIFB in each of those settings, adjusted to take account of stockout rates and potency of uterotonics. This exercise was conducted in 2013 in Mozambique, Tanzania, the state of Jharkhand in India, and Yemen.

**Results:**

Available data showed that deliveries in public health facilities account for approximately half of births in Mozambique and Tanzania, 16% in Jharkhand and 24% of births in Yemen. Significant proportions of births occur in private facilities in Jharkhand and faith-based facilities in Tanzania. Estimated uterotonic use for facility births ranged from 70 to 100%. Uterotonics are not used routinely for PPH prevention at home births in any of the settings. National UUIFB coverage estimates of all births were 43% in Mozambique, 40% in Tanzania, 44% in Jharkhand, and 14% in Yemen.

**Conclusion:**

This methodology for estimating coverage of UUIFB was found to be feasible and acceptable. While the exercise produces imprecise estimates whose validity cannot be assessed objectively in the absence of a gold standard estimate, stakeholders felt they were accurate enough to be actionable. The exercise highlighted information and practice gaps and promoted discussion on ways to improve UUIFB measurement and coverage, particularly of home births. Further follow up is needed to verify actions taken. The methodology produces useful data to help accelerate efforts to reduce maternal mortality.

## Background

Globally, the number of maternal deaths has significantly declined, from an estimated 376,034 in 1990 to 292, 982 in 2013 [1]. The rates of change however are not sufficient to achieve a reduction in maternal mortality ratio by three-quarters by 2015 [1]. To accelerate the decline in countries where the largest numbers of deaths occur, the major causes of maternal mortality need to be addressed. Postpartum hemorrhage (PPH) is the leading cause in developing countries, and contributes nearly 20% of maternal deaths globally [2].

For the prevention of PPH, a uterotonic should be given to all women in the third stage of labor (i.e., immediately after birth), preferably oxytocin [3]. Prophylactic uterotonic use remains the most effective of the three components of active management of the third stage of labor (AMTSL). About 38% of women in developing countries who do not deliver with a skilled birth attendant [4] are not offered oxytocin or AMTSL. Misoprostol is an alternative uterotonic, and it has been used safely for PPH prevention at home births [5].

Numerous countries and development agencies have committed substantial resources to improving the coverage and quality of uterotonic use for PPH prevention, but lack a widely reported standardized indicator to monitor the use of prophylactic uterotonics. Current priority maternal health indicators [6] focus on contacts with the health system (e.g., percentage of births with a skilled attendant), but do not measure the content of care provided. The World Health Organization therefore recommends that a content-based indicator be used, defined as the percentage of women that receive a prophylactic uterotonic drug after birth among all women giving birth. Currently, data on prophylactic uterotonic use for all women giving birth are scarce. A 2012 survey conducted by the Maternal and Child Health Integrated Program found that only 15 of 37 USAID MNCH priority countries had an indicator for AMTSL and not all of these reported it to the national level [7]. None reported uterotonic coverage for women delivering at home.

To be able to measure this new content-based indicator for this key practice and track progress over time on prophylactic uterotonic use for all births, an exercise was developed to help national experts and stakeholders estimate national population-level coverage. For the purposes of this paper, this indicator is referred to “uterotonic use immediately after birth (UUIFB)” and is defined as 10 IU IM oxytocin or 600 mcg of misoprostol PO, given in the third stage of labor at any birth (i.e., at home or in a health facility). This article reports the UUIFB estimates derived from the exercise conducted in four settings: Mozambique, Tanzania, the state of Jharkhand, India, and Yemen.

## Methods

### Development of the methodology

A landscape analysis was conducted in mid-2012 to identify current and potential methods of estimating UUIFB nationally. Key findings were the scarcity of data on UUIFB and the need to improve estimation of this key intervention. Table 1 presents six possible methods for UUIFB estimation, roughly ranked for validity (i.e., accuracy). Generally, the methods that produce the most valid information are the more expensive and time-consuming to conduct. All methods are exclusively or mainly focused on facility-based deliveries and are unlikely to capture UUIFB at home births.

**Table 1:** **Possible methods for estimating national UUIFB coverage**

**Method (ranked by validity)**	**Feasibility and validity considerations**	**Example**
1 Observational assessments of quality of care	● Most accurate information for those births observed	Done in MCHIP Quality of Care assessments and now included as an optional Demographic and health Survey (DHS) Service Provision Assessment (SPA) module (done in Kenya; planned for Malawi)
	● Not commonly done	
	● Expensive to conduct	
	● When done, not likely to be on a large and nationally representative sample	
	● Likely excludes home births	
2 Facility readiness assessments	● Need to extrapolate from availability of commodity/personnel to actual use of uterotonic	DHS SPA, Service Availability and Readiness Assessment (World Health Organization)
	● Expensive to conduct on a representative sample on a regular basis	
	● Likely excludes home births	
3 Routine Health Management Information System (HMIS) data	● Only possible where data are recorded in registers and reported to higher levels	Included in registers in some countries (e.g., Mozambique)
	● HMIS data quality variable	
	● No additional data collection costs required	
	● May not include community-level reporting on home births	
4 Data from sentinel surveillance sites	● Only possible where such sites are available	MCHIP used this method in Kenya (results unpublished)
	● No additional data collection costs required	
	● Question of generalizability	
5 Extrapolation from service contact data	● Estimates require extrapolation with questionable assumptions (i.e., that skilled birth attendant and/or institutional birth implies use of uterotonic in most or all of covered births).	Suggested method – expert panel not aware of previous experience with this
	● No additional data collection costs required	
6 Survey of key informants	● Easy and low cost to interview individuals or group of informants	Suggested method – expert panel not aware of previous experience with this.
	● Likely to be subjective with opinions likely biased and/or based on incomplete information	

In December 2012 MCHIP presented the results of the landscape analysis to group of 19 experts and researchers working in international maternal health programs. The global expert panel confirmed the value of the process given that such estimates are not currently available. It reviewed possible methods of estimation (Table 1) and recommended as a rapid estimation exercise combining several methods to balance validity with feasibility considerations. First, the best available data from all available sources (i.e., the results of all methods encompassed by Methods 1–5 in Table 1) would be collected and evaluated for data quality by an in-country facilitator, preferably someone with a strong research background. Next, the Ministry of Health (MOH) would convene a panel of 15–20 experts with in-depth knowledge and experience with maternal health measurement and practice, such as senior MOH officials, researchers, representatives from technical organizations (e.g., WHO, UNICEF, donors, international and national non-governmental organizations) and leaders from professional associations. The panel would be surveyed (i.e., an example of Method 6 in Table 1) and then invited to a meeting to review the gathered information and use the algorithm described below to generate a national estimate of UUIFB coverage.

The algorithm used three data elements focusing on coverage in all birth locations, weighted by the percent of women giving birth in each of those locations. Information was gathered from publically available health management information systems (HMIS) or surveys or program data. When such data were not available, estimates were derived through panel deliberation and consensus based on the experience of the panel members themselves:

1. Distribution of birth locations
  ○ Proportion of facility-based births by sector: public, private, and non-governmental organization (NGO). Further stratification by health facility level if deemed relevant to the calculation by the panel (e.g. significantly different UUIFB coverage at hospitals compared to health centers).
  ○ Proportion of home births, disaggregated by attendance of a skilled provider
2. Measurements or estimates for the coverage of UUIFB using a recommended uterotonic (i.e., oxytocin, ergometrine or misoprostol) in each identified birth location.
3. Adjustments to those initial measurements or estimates of UUIFB, considering additional information on frequency of stockouts and uterotonic quality.

The group also considered supporting qualitative information derived from relevant documents, including national policies on uterotonic use, providers authorized to use uterotonics, presence of uterotonics on the List of Essential Medicines.

The quantitative information was utilized to construct a four-term equation applied to each relevant birth location and then summed over all those birth locations:

$$ \mathbf{UUIF}{\mathbf{B}}_{\mathrm{national}}=\Sigma \left({\mathrm{P}}_{\mathrm{a}}*\mathrm{E}\mathrm{s}{\mathrm{t}}_{\mathrm{a}}*\mathrm{S}{\mathrm{I}}_{\mathrm{a}}*{\mathrm{Q}}_{\mathrm{a}}\right)+\left({\mathrm{P}}_{\mathrm{b}}*\mathrm{E}\mathrm{s}{\mathrm{t}}_{\mathrm{b}}*\mathrm{S}{\mathrm{I}}_{\mathrm{b}}*{\mathrm{Q}}_{\mathrm{b}}\right)+\dots \left({\mathrm{P}}_{\mathrm{z}}*\mathrm{E}\mathrm{s}{\mathrm{t}}_{\mathrm{z}}*\mathrm{S}{\mathrm{I}}_{\mathrm{z}}*{\mathrm{Q}}_{\mathrm{z}}\right) $$

Where:

UUIFB_national_ is the national estimate of UUIFB coverage

P_a_ is the proportion of births in Location A (e.g., home, public facility, etc.)

Est_a_ is the initial estimate of UUIFB coverage in Location A

SI_a_ is the “stock-in” rate of uterotonic in Location A (i.e., commodity availability or 100% minus the stock-out rate)

Q_a_ , *an optional factor,* is the estimated quality (i.e., biological potency) of the uterotonic in Location A

Σ indicates that this calculation is performed for all identified birth locations (i.e., Locations A to Z).

### Country selection

Four settings were purposively selected. The settings, which cover a variety of geography and contexts, had MOH interest and were programmatically important because of large numbers of maternal deaths. Maternal mortality ratio estimates in the four settings are high (i.e., 270–480 per 100,000 live births) [8]. A single Indian state was selected because of the heterogeneity of health service delivery across India and the fact that authority is decentralized to states for health programs.

### Implementation of the estimation exercise

#### General description and preparation

The exercises were conducted in Mozambique, Tanzania and Jharkhand, India between April and July 2013 and in Yemen December 2013. The approach was the Modified Delphi method [9-11] led by a small group of in-country facilitators to help the expert group reach a consensus on the national coverage estimate. First, before convening the expert group, background information was collected and reviewed on location of births, policies and guidelines related to uterotonic use, the national List of Essential Medicines, peer-reviewed literature and program reports on availability of uterotonics, and any other information relevant to UUIFB coverage estimates. Supplemental information on providers sanctioned to provide uterotonics following birth, types of uterotonics in use, and the existence and coverage of community-based misoprostol distribution for home births also were gathered. HMIS reports, Demographic and Health Surveys (DHS), and/or Multiple Indicator Cluster Survey (MICS) reports, peer-reviewed literature, health facility assessments and other relevant documents were collected and reviewed.

A national expert panel of 15 to 30 professionals was called to a one-day meeting. The panel composition varied between countries, but was always comprised a diverse set of experts that included MOH officials working on maternal and newborn health services, senior members of national professional associations, representatives from private sector associations, key maternal health academics and researchers, experts in commodity supplies, facility-based service providers, and stakeholders familiar with prevailing practices during home birth (see Table 2). Before the exercise, participants were sent a brief survey to gather key information on delivery locations, national policies, guidelines and practices. The consultation with the expert panel was a facilitated three step process, described below.

**Table 2:** **Number and description of experts participating in each country panel**

**Setting**	**Senior MOH officials**	**Key maternal health academics and researchers**	**Representatives from technical organizations**	**Leaders from professional associations**	**Facility-based service providers**	**Stakeholders familiar with prevailing practices during home birth**	**Total**
Mozambique	1	1	7	2	2	1	**14**
Tanzania	0	4	10	4	2	0	**20**
Jharkhand, India	6	3	12	1	4	5	**31**
Yemen	1	3	6	2	7	3	**22**

#### Step 1

The expert panel first reviewed available evidence on the location of births in the country from the most recent DHS. Because no DHS had been conducted in Jharkhand or Yemen, a similar population-based survey was used for these estimates [12,13]. The group then reviewed HMIS data for further stratification by level of service delivery (i.e., births at regional hospitals or lower level health facilities) if it was deemed relevant to UUIFB provision.

#### Step 2

Beginning with data sources considered most valid and representative (as described in Table 1), the panel then discussed initial estimates of UUIFB in each birth location identified in Step 1. The panel considered all available data, which had been gathered by the facilitator in consultation with panel members. The data sources ranged from observational studies of quality (Mozambique and Tanzania for public facilities), to HMIS data (all but Yemen), to opinion of the panel members when no other data source was available, as was usually the case for non-public sector facilities. Variations on measurements around timing of the provision of uterotonic reported in the available data sources (e.g., within one versus three minutes after birth) were discussed, and a consensus decision was made about which measurements would be used on a country-by-country basis.

#### Step 3

Finally, the panel considered factors that might require adjustment of the initial estimates of UUIFB in each birth location, such as stock-out rates and reports on drug quality or potency. The modifying factors were used to adjust the initial estimate. The qualitative information (i.e., on providers authorized to use uterotonics, policies, and presence of the uterotonics on the List of Essential Medicines) was used as a check on the likely accuracy of quantitative estimates.

#### Final calculation and recommendations

Table 3 summarizes all the data sources used for the calculation. The calculation of the UUIFB value was done by the expert panel, facilitated by use of a spreadsheet that calculated the weighted average. Upper and lower bounds on the estimate then were assigned based on a consensus view of the uncertainty of various data elements included in the calculation. Panel members discussed key gaps in information, likely causes of lower than expected coverage and recommendations for improving UUIFB coverage and its estimation.

**Table 3:** **Data sources used for parameters needed to estimate UUIFB coverage**

	**Mozambique**	**Tanzania**	**Jharkhand**	**Yemen**
**% Births in location (P)**	Mozambique National Statistics Institute and ICF International. 2011. *Mozambique Demographic and Health Survey*. Calverton, MD, USA	National Bureau of Statistics (NBS) [Tanzania] and ICF Macro. 2011. *Tanzania Demographic and Health Survey* 2010. Dar es Salaam, Tanzania: NBS and ICF Macro.	Vital Statistics Division, Office of the Registrar General & Census Commissioner, Government of India: *Annual Health Survey 2010–11 Fact Sheet Jharkhand*. New Delhi, 2012.	Ministry of Planning and International Cooperation, Republic of Yemen: *National Social Protection Monitoring Survey of Yemen: Baseline Analytical Report June 2013*. Brasilia, 2013.
**% UUIFB in location (Est)**	***Public facilities:*** Mozambique Ministry of Health and MCHIP. 2011. *Quality and Humanization of Care Assessment: A Study of the Quality of Maternal and Newborn Care Delivered in Mozambique’s Model Maternities*. Accessed April2013: http://www.mchip.net/node/1847.	***Public facilities:*** MCHIP: *Quality of Care for Prevention and Management of Common Maternal and Newborn Complications: A study of 12 regions in Tanzania. Report 2: Findings on Labour, Delivery and Newborn Care* Washington DC, 2013. http://www.mchip.net/sites/default/files/mchipfiles/Tanzania_%20QoC_StudyReport_FINAL_0.pdf	Consensus opinion of Expert Panel, based on direct experience as clinicians and managers in each of the settings.	Consensus opinion of Expert Panel, based on direct experience as clinicians and managers in each of the settings.
**% stock-in (SI)**	***Public facilities:*** Ministry of Health, Republic of Mozambique and UNFPA: *Second Survey of Availability of Modern Contraceptives and Essential Lifesaving Maternal/Reproductive Health Medicines in Service Delivery Points. Report of Mozambique 2011*. Maputo, 2012	*All facilities:* Stanton C, Armbruster D, Knight R, Ariawan I, Gbangbade S, Getachew A, Portillo JA, Jarquin D, Marin F, Mfinanga S, Vallecillo J, Johnson H, Sintasath D: Use of active management of the third stage of labour in seven developing countries. *Bull World Health Organ*. 2009 Mar;**87**(3):207–15.	Consensus opinion of Expert Panel, based on direct experience as clinicians and managers in each of the settings.	Consensus opinion of Expert Panel, based on direct experience as clinicians and managers in each of the settings.
	***Private facilities:*** Expert panel consensus			

MCHIP also reconvened the global expert panel in July 2013 to review the methodology in light of results from the initial three exercises.

### Ethical review

No IRB approval was required because it was not human subjects research, gathered no primary data, and used only publically available data.

## Results

### Estimates of distribution of births by location

**Figure 1:**
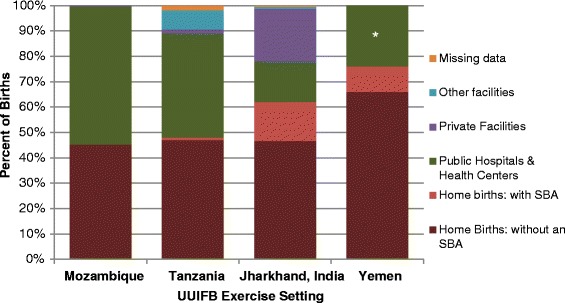
**Estimates of distribution of births by location in each setting.** Dark red represents Home births without a skilled birth attendant (SBA); pink represents Home births with a skilled birth attendant; green represents births in public hospitals and health facilities; purple represents births in private health facilities; blue represents other health facilities; orange represents missing data.

Figure 1 presents the distribution of births by delivery location in the four settings, generated by the expert panels [14,15]. In Tanzania and Mozambique, home births account for close to half of all deliveries (48.1 and 45.2%, respectively), while in Jharkhand they account for 62.0%, and 76.0% in Yemen. Facility-based deliveries in public hospitals and health centers account for 54.6% of births in Mozambique, 41.0% in Tanzania, and 16.0% in Jharkhand. In the case of Mozambique, facility deliveries outside the public sector were negligible (0.2%), but in other settings, a substantial fraction of births occur outside the public sector, as in Jharkhand (20.8% in private; 0.8% in faith-based NGO) and Tanzania (7.5% in faith-based NGO facilities). Data from public and private facilities are combined in Yemen.

Because of known differences in coverage of UUIFB in different levels of public health facilities in some settings and for births attended by skilled birth attendants (versus non-skilled attendants), some refinements were applied to the estimates of distribution of birth locations and these are shown in Figure 1. In Tanzania, public facility births were further stratified by level (health center versus hospital). A small fraction of home births are attended by a skilled birth attendant (SBA) in three of the settings (Tanzania, Yemen and Jharkhand); and a small percentage (0.2%) of facility-based deliveries in public facilities are conducted by non-qualified personnel in Jharkhand.

### Initial UUIFB coverage estimates by delivery location

All available information was reviewed and discussed to arrive at UUIFB coverage for each identified delivery location. Data from facility-based assessments were used where available, complemented by HMIS data. In Yemen, no specific data on UUIFB in any location were available, so the expert panel made estimates, based on its consensus opinion.

For facility-based birth locations, initial UUIFB coverage estimates ranged from a low of 70% in public facilities in Yemen to as high as 100% for private facility births in Jharkhand and Mozambique. In Mozambique and Tanzania, recent observational assessments of the quality of labor and delivery care had been conducted, from which the panel was able to draw facility-based UUIFB provision [16,17]. Based on these data the Tanzanian panel estimated UUIFB coverage in most public and private facilities to be 81.5%, averaging findings from health centers (71%) and central hospitals (99%). The Mozambique panel took the national HMIS reported uterotonic use (94%), and revised this figure downward based observational assessment finding that uterotonic use within three minutes of birth (i.e., an even more stringent standard than UUIFB implies) was 68%. In Yemen, UUIFB coverage was estimated to be 70% for facility deliveries. In Jharkhand UUIFB coverage was estimated to be 90% for all public and private facilities.

For home births, little information on UUIFB coverage was available. In Jharkhand approximately 15% of deliveries occur at home with an SBA, and the panel’s consensus opinion was that UUIFB coverage in this setting was approximately 85%. Taking into account the distribution of births and UUIFB in this delivery location, UUIFB coverage for home births attended by an SBA contributed 29.8% of total UUIFB coverage in Jharkhand and 42.9% in Yemen, but in Tanzania it contributed only 2.0% and in Mozambique 0%. In all settings, home-based deliveries without an SBA were estimated as having 0% UUIFB coverage. This reflected the absence of home-based uterotonic provision programs, such as distribution of misoprostol for non-SBA attended home births.

### Adjustments to initial coverage estimates by birth location

Stockouts of uterotonic were a key modifier applied to the original coverage estimate.

Estimated stockouts of uterotonic for Jharkhand ranged from 0-33%, with the opinion of the panel that stockouts were more frequent at more peripheral facilities. Stockouts at all facilities in Tanzania were estimated at 2%, taking facility audit findings into consideration. In Mozambique, a 2011 national UNFPA commodity availability assessment showed a nationwide stockout rate of 2.5% across all types and levels of facilities, both public and private [18]. The expert panel in Yemen calculated a uterotonic access rate, which factored in stockouts as well as other supply/access issues, for SBAs at home births at 90% and 50% for facility births. This is shown in Table 4.

**Table 4:** **Calculation of UUIFB coverage estimates for Mozambique, Tanzania, Jharkhand (India), and Yemen**

	**Mozambique**				**Tanzania**				**Jharkhand**				**Yemen**			
**Birth location**	**% Births in location (P)**	**% UUIFBin location (Est)**	**% stock-in (SI)**	**Contribution to national coverage**	**% Births in location (P)**	**% UUIFB in location (Est)**	**% stock-in (SI)**	**Contribution to national coverage**	**% Births in location (P)**	**% UUIFB in location (Est)**	**% stock-in (SI)**	**Contribution to state coverage**	**% Births in location (P)**	**% UUIFB in location (Est)**	**% stock-in (SI)**	**Contribution to national coverage**
**Home births: without an SBA**	45.2%	0%	─	0.0%	47.0%	0%	─	0.0%	46.7%	0%	─	0.0%	66.0%	0.0%	─	0.0%
**Home births: with SBA**	─	─	─	─	1.1%	70%	N/A	0.8%	15.3%	85%	100%	13.0%	10.0%	70.0%	90.0%^e^	6.3%
**Public facilities**	54.6%^a^	80% (62-94%)	97.5%	42.6%	41.0%	71%^f^	98%	30.9% (28.7-32.8%)	16.0%^c^	90%	67-93%^d^	11.8%	24.0%	70.0%^b^	50.0%^e^	8.4%^b^
						81.5%^f^										
						99%^f^										
**Private facilities**	0.2%	100%	100%	0.2%	1.6%	81.5% (80-83%)	98%	1.3% (1.2-1.3%)	20.8%	90%	100%	18.7%	─	─	─	─
**Other facilities (Faith-based, NGO)**	─	─	─	─	7.5%	81.5% (80-83%)	98%	6.0% (5.9-6.1%)	0.8%	N/A	N/A	N/A	─	─	─	─
**Missing data**	─	─	─	─	1.8%	--	N/A	1.3%	0.4%	─	─	─	─	─	─	─
**National UUIFB coverage estimate**	100.0%			43% (34–49%)	100.0%			40% (37–42%)	100.0%			44%	100.0%			15%

N/A not available, ─ not applicable.

^a^Since there are not separate coverage or stockout data for Hospitals and Health Centers, the two are reported together.

^b^Data from Yemen combines public and private facility delivery data.

^c^The expert panel in Jharkhand had disaggregated facility deliveries by SBA (15.8%) and non-SBA (0.2%).

^d^The expert panel in Jharkhand had disaggregated facility deliveries by three types of health facilities and attributed different stock-in rates for each, therefore a range is presented and relative stock-in rates reflected in the calculation of state coverage.

^e^The expert panel in Yemen calculated a uterotonic access rate which considered stock in facilities, availability in outside pharmacies, ability to pay and frequency with which facilities provide it to women who cannot afford to purchase the drugs outside.

^f^The expert panel in Tanzania disaggregated UUIFB estimations by health center/dispensary level, district/regional hospitals and central hospitals.

**Figure 2:**
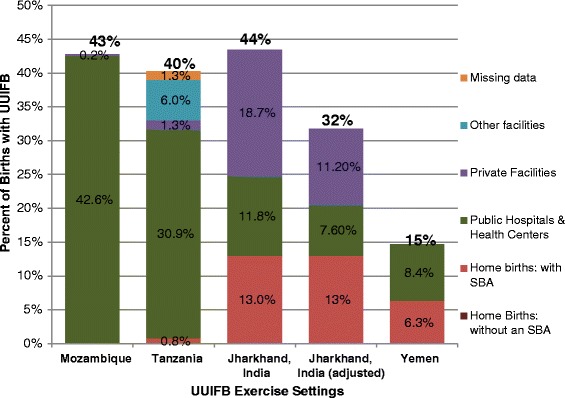
**Final consensus estimate of total UUIFB coverage.** Dark red represents Home births without a skilled birth attendant (SBA); pink represents home births with a skilled birth attendant; green represents births in public hospitals and health facilities; purple represents births in private health facilities; blue represents other health facilities; orange represents missing data.

The Jharkhand expert panel incorporated an assumption that 40% of oxytocin samples were not potent because of poor storage, based on findings from a recent assessment at health facilities and took this into account in their estimation of the effective coverage rate of quality uterotonic. This adjustment was not done in the other three settings. The final estimates of coverage of UUIFB among all births were 15% in Yemen, 40% in Tanzania, 43% in Mozambique, and 44% (unadjusted) or 32% (quality adjusted) in Jharkhand. Figure 2 presents the contributions from each setting to the national coverage estimate and Table 4 summarizes the data elements used for calculation of UUIFB in the four settings. Table 5 presents the key recommendations and actionable outcomes from each exercise.

**Table 5:** **Recommendations by the expert panel from the UUIFB estimation exercise in each setting**

**Setting**	**Key recommendations**
**Jharkhand, India**	● Promote awareness among women and families about the potential and correct use of misoprostol for prevention of PPH.
	● Develop a program for the advanced distribution of misoprostol to women who deliver at home.
	● Improve commodity management to reduce the rate of stock-outs of uterotonic drugs.
	● Further understand and improve the quality of oxytocin.
	● Improve data gathering and data quality for UUIFB.
**Mozambique**	● Improve UUIFB coverage in the public sector through quality improvement measures.
	● Expand the use of misoprostol in the community. Just expanding to the 35 planned districts this year would increase national UUIFB coverage by more than 10%.
	● Emphasize in maternity norms that oxytocin should be given within one minute of birth. The birth attendant must prepare the dose before the birth.
	● The MOH should authorize all providers who attend births to give oxytocin.
	● Given its importance as a medicine, the need is urgent to investigate the potency of oxytocin.
	● MCHIP could make funds available to finance the purchase of equipment to strengthen the cold chain for oxytocin.
**Tanzania**	● Improve data on home-based use of uterotonics.
	● Improve commodity management and tracking, especially at lower level health facilities
	● Track stockout of all possible approved uterotonics, rather than tracking them individually
	● Improve data quality and gathering on UUIFB, including defining UUIFB for these purposes
**Yemen**	● Increase supply of uterotonics in facilities
	● Increase knowledge of providers about uterotonic use
	● Develop educational materials to clarify providers’ understanding of the benefits and uses of misoprostol
	● Pilot the use of misoprostol for prevention of PPH at home birth
	● Review/modify the job description of midwives to ensure permission to use misoprostol for PPH prevention
	● Work with High Commission for Medications to approve the use of misoprostol for PPH prevention and treatment.

## Discussion

This new methodology was effective in generating a national-level estimate of UUIFB coverage in all four settings and therefore filled the need for a content-based PPH-related indicator within national safe motherhood programs. Although there currently is no way to validate the findings, the exercise produced results that were acceptable to the expert panel. It provided the MOH with measurement for monitoring and planning, as well as actionable information to address gaps in data collection, data quality and service provision.

PPH remains the leading cause of maternal death in most developing countries. AMTSL substantially reduces the incidence of severe PPH [19], and more recent evidence concludes that use of a uterotonic is the most effective component of AMTSL [20,21]. AMTSL has been widely promoted in skilled attendance at birth particularly at health facilities. At the same time, at least 19 countries have introduced or expanded distribution of misoprostol as a PPH prevention strategy at home births [22]. National safe motherhood programs, however, do not have data on national UUIFB coverage for all births to inform planning and evaluation because data on this intervention are not easily measured on a population basis. Health facility readiness assessments done on a nationally representative sample of facilities (e.g., Service Provision Assessments, Service Availability and Readiness Assessments, and the Averting Maternal Death and Disability assessments) are conducted occasionally and typically measure inputs (such as the availability of uterotonics) and not actual practice. Facility assessments using observation of labor and delivery practices produce the most valid data, but are even less commonly done, especially on a nationally representative sample of facilities. One such observational assessment, conducted mainly in district and referral hospitals in seven countries in Asia, Africa and Latin America from 2005–2006, found that almost all (98–100%) women received a uterotonic during the third or fourth stage of labor [23]. Few national HMIS currently record and report data on AMTSL or UUIFB. Data from all three of these sources often are limited to public sector facilities and do not take account of the substantial proportion of women who give birth at home. Population-based surveys such as DHS and MICS rely on self-reported data and therefore do not produce reliable information on the coverage of UUIFB, as a recent study has shown that women’s recall about the receipt of an injection immediately following birth is very poor [24].

The uterotonic estimation exercise described in this paper was devised to fill this information gap on UUIFB coverage. The method is similar to that used for the Maternal and Neonatal Program Effort Index to estimate national programmatic effort in the reduction of maternal and newborn death [25] and the method has also been used to estimate the effectiveness of maternal and newborn interventions on mortality [10,11]. Participants in several settings stated that they felt the process was feasible and transparent and helped them arrive at an estimate accurate enough to be actionable. For example, the panels identified access to uterotonics as the largest contributor to suboptimal national UUIFB coverage. Because the methodology estimates population coverage, it should allow Ministries of Health to be more strategic in identifying the most potentially effective strategies to improve UUIFB coverage for all births regardless of delivery location, and thereby promote more equitable uterotonic coverage.

The exercise highlighted how uterotonic availability and quality can influence national UUIFB coverage. Yet uterotonic availability at the facility level is not tracked nationally in any of the four settings. In Tanzania, the national program tracks oxytocin, ergometrine and misoprostol stockouts separately, but does not identify when facilities experience a stockout of all uterotonics simultaneously. Degradation of injectable uterotonics due to exposure to light and heat has been documented [26], and recent studies of uterotonics collected from health facilities and pharmacies in developing countries have raised concerns about quality, particularly of oxytocin [27-29]. A study in India found 22%-50% of oxytocin and 44-54% of methylergometrine samples did not meet manufacturer specifications for potency (90%-110% active pharmaceutical ingredient) in Uttar Pradesh, a neighboring state to Jharkhand. [Personal Communication, Nitya Nand Deepak, PATH/India] Concerns persist as well about misoprostol deterioration due to moisture, if not packaged properly [30]. Clearly, efforts to raise effective coverage can be blunted by inattention to pharmaceutical quality. This should be taken into account as national governments formulate strategies to raise UUIFB coverage rates.

This exercise also highlighted the programmatic gaps around UUIFB for home births. Although WHO recommends misoprostol for PPH prevention at home births where SBAs and oxytocin are not available [4], none of the four settings studied in this exercise currently have a large-scale program to ensure UUIFB for the nearly 50% of births occurring at home. In the short term, national UUIFB coverage in all four settings can only substantially increase if UUIFB at home births is addressed. Evidence suggests that community-based programs for PPH prevention at home births using misoprostol achieve the greatest coverage through advance distribution by community health agents at home visits and self-administration [22].

Because of the nature of the exercise, estimates are dependent on the quality of data available. Issues with data quality and gaps became evident through the exercise in all four settings. An important activity of the exercise was advocacy for improved data collection and quality related to UUIFB, similar to experiences from data-use workshops [31]. Dissemination of the data may stimulate greater attention to measurement of this key indicator and hence better future estimates. The rapidity of the exercise allows for quick input into program planning and makes possible repeated measurements. Since the panels developed a list of priority interventions to improve UUIFB coverage and its measurement, these repeat measures could be used to track progress.

### Limitations

This exercise was designed to provide for the first time population-level estimates in a field with a notable lack of information. To do this, the panels made educated judgments about divergent estimates for some parameters and made rough estimates of others in order to arrive at its final national coverage figure. The data sources that were considered by the expert panels ranged from population-based surveys (for birth location), to observational studies of service delivery, data from HMIS, and self-reported provider practice. In a few cases where no systematic information existed, expert opinion was used. There is no gold standard methodology for comparison. Given that there are no estimates for comparison, the validity of the UUIFB estimates cannot be objectively assessed. The figures on UUIFB coverage for some birth locations were the least precise data element with the widest possible range. The uncertainty in the estimation of this parameter was highlighted in those instances in which more than one estimate was available, as these were sometimes divergent. None of the exercises had data from current, nationally representative studies on uterotonic use after birth, and thus had to extrapolate findings from studies not meant for this purpose. Expert panels also used their own judgment in assigning weight to data of varying levels of quality and scope, with facilitation guidance from experts on measurement issues. Some of the major extrapolation challenges the panels faced included measurements of the use of different types of uterotonic separately; measurements which quantified uterotonic use in a timeframe that did not correspond exactly to the concept of UUIFB (e.g., one minute after birth or three minutes after birth); and use of data from small and/or old studies. This methodology should be seen as an interim step toward full integration of indicators on uterotonic coverage, both for facility and home birth. There should be follow-up to encourage progress programmatically and in collecting more complete and accurate information.

## Conclusions

The UUIFB coverage estimation exercise produced estimated levels of coverage in all four settings that were much lower than desired or anticipated by many panel members. While the validity of the estimates is untested because there is no gold standard for comparison, the exercise fills an important gap in monitoring of maternal health programs by measuring intervention coverage of all births. The exercise raised awareness among national experts of low levels of UUIFB and provided them with information for monitoring and planning that was previously unavailable. Participants felt the process was feasible, transparent and accurate enough to be actionable. It highlighted critical gaps in the measurement of uterotonic provision. It is recommended in settings where PPH is a significant cause of maternal mortality and PPH prevention interventions are being implemented. UUIFB coverage estimates can provide useful data to help plan efforts to accelerate national and global efforts to reduce maternal mortality. Similar estimation exercises could be devised for other key maternal health interventions for which information is also currently lacking – for instance, use of magnesium sulfate for cases of eclampsia and severe pre-eclampsia.

## Abbreviations

AMTSL: Active management of the third stage of labor
DHS: Demographic and health survey
HMIS: Health management information system
MCHIP: Maternal and Child Health Integrated Program
MICS: Multiple Indicator Cluster Survey
MOH: Ministry of Health
NGO: Non-governmental organization
PPH: Postpartum hemorrhage
SBA: Skilled birth attendant
UNFPA: United Nations Population Fund
UUIFB: Uterotonic use immediately following birth
USAID: United States Agency for International Development
WHO: World Health Organization
